# “My motivation was to save”: a qualitative study exploring factors influencing motivation of community healthcare workers in a cervical cancer screening program in Dschang, Cameroon

**DOI:** 10.1186/s12978-022-01420-y

**Published:** 2022-06-06

**Authors:** Pauline Hämmerli, Alida Datchoua Moukam, Ania Wisniak, Jessica Sormani, Pierre Vassilakos, Bruno Kenfack, Patrick Petignat, Nicole Christine Schmidt

**Affiliations:** 1 Faculty of Medicine, University of Geneva, Geneva, Switzerland; 2 Department of Gynaecology and Obstetrics, District Hospital of Dschang, Dschang, Cameroon; 3 Gynaecology Division, Department of Paediatrics, Gynaecology and Obstetrics, University Hospitals of Geneva, Geneva, Switzerland; 4 Geneva School of Health Sciences, HESSO University of Applied Sciences and Arts Western Switzerland, Geneva, Switzerland; 5 Geneva Foundation for Medical Education and Research, Geneva, Switzerland; 6 Faculty of Social Science, Catholic University of Applied Science, Preysingstr. 95, 81667 Munich, Germany

**Keywords:** Community healthcare workers, Motivation, Retention, Cameroon, Cervical cancer, Cervical cancer screening program, Qualitative study

## Abstract

**Background:**

Cervical cancer is a major public health issue among women in Cameroon and uptake of screening programs remains a challenge in many low- and middle-income countries. Community healthcare workers can play an important role in promoting cervical cancer services. This study aimed to explore factors affecting the motivation of community healthcare workers in a cervical cancer screening program in Dschang, Cameroon.

**Methods:**

A qualitative approach including 11 in-depth individual interviews with community healthcare workers was used. The interviews were audio-recorded, transcribed and coded using thematic analysis assisted by ATLAS.ti software.

**Results:**

Four women and seven men aged between 21 and 77 years old were interviewed. Community healthcare workers had high motivation. Factors affecting motivation were divided into individual and environmental level, based on a theoretical framework. Factors with a positive influence on motivation were mainly on the individual side while impeding factors were mainly associated with the environmental level.

**Conclusions:**

Key interventions to improve motivation among community healthcare workers include: (i) more training and supportive supervision; (ii) evaluation of remuneration systems by workload; and (iii) provision of job-enabling resources such as uniforms, cellphone cards and transport.

*Trial registration:* Geneva Cantonal Ethics Committee on Human Research (No. 2017-01110) and the Cameroonian National Ethics Committee for Human Health Research (No. 2018/07/1083/CE/CNERSH/SP).

## Plain Language Summary

Cervical cancer is the second leading cause of cancer-related death among women in Cameroon. This is mainly due to the absence of routine cervical cancer screening programs and the lack of treatment. However, even in case of availability of cervical cancer screening programs, barriers exist. Therefore, community healthcare workers have been involved successfully to increase the screening coverage. As retention of these actors remains a challenge, the main objective of this study was to identify key factors affecting their motivation aiming to suggest interventions to address motivational factors. 11 qualitative interviews with individual community healthcare workers were conducted at a cervical cancer screening program in Dschang, Cameroon. Motivation was generally high and affecting factors were divided into the individual and the environmental level. Factors with a positive influence on motivation were mainly on the individual side while impeding factors were mainly associated with the environmental level. Ongoing collaboration with community healthcare workers is beneficial for cervical cancer screening programs, and key interventions should therefore include: (i) more training and supportive supervision; (ii) evaluation of remuneration systems by workload; and (iii) provision of job-enabling resources such as uniforms, cellphone cards and transport.

## Introduction

Cervical cancer is the fourth most frequent cancer worldwide among women [1]. Nearly 85% of new cases and approximately 90% of related deaths occur in low- and middle-income countries like Cameroon [2]. Persistence of a high-risk human papillomavirus (HPV) infection is the major cause of both precancerous lesions and cervical cancer [3]. HPV screening and vaccination are therefore the main forms of preventive healthcare [4]. Cervical cancer is both preventable and curable if detected early and treated appropriately. However, disparities in incidence and mortality rates between countries with low and high resource levels persist, mainly due to limited access to systematic screening and vaccination and lack of appropriate diagnostics and treatment [5, 6]. In May 2018, the World Health Organization (WHO) therefore launched a global call to accelerate the elimination of cervical cancer. In August 2020, the 73rd World Health Assembly adopted the Global Strategy for its elimination [7]. Each country is expected to reach the 90-70-90 targets by 2030 [7] (see Box 1).

**Box 1:** Global strategy of the World Health Organization to eliminate cervical cancer as a public health problem

Global strategy to accelerate the elimination of cervical cancer 2020–2030	
*Vaccination*: 90% of girls to be fully vaccinated with the HPV vaccine by the age of 15; *Screening*: 70% of women to be screened using a high-performance test by the age of 35, and again by the age of 45; *Treatment*: 90% of women with pre-cancer treated and 90% of women with invasive cancer managed	

The second objective of the WHO’s global strategy sets out that countries should achieve a 70% coverage rate for cervical cancer screening by 2030. However, by 2020, only 18% of countries providing screening for cervical cancer had reached this target [8]. Traditionally, cervical cancer screening has mainly been cytology-based, which causes difficulties in low- and middle-income countries because of inadequate health infrastructure, lack of trained professionals and economic hardship [9]. Alternative methods have therefore been developed, including the WHO’s recommended test–triage–treat (3T) strategy, which can be performed in a single visit [9–11]. The first step in this method is a test for high-risk HPV types using self-sampling. This is followed for HPV-positive women by a triage with visual inspection of the cervix after application of acetic acid and Lugol’s iodine, and then treatment with thermal ablation if required. The procedure is completed with a follow up of HPV-positive women [12–14].

Technology and techniques for cervical cancer screening are improving. However, the uptake of programs is affected by factors including socio-economic issues, structural issues (such as distance to facilities and availability of transport) and sociocultural factors (such as psychological such as “fear of the results” or knowledge related barriers) [15, 16]. Community healthcare workers (CHWs) can play an important role in overcoming these obstacles. The 72nd World Health Assembly therefore adopted a resolution: “highlighting the role of CHWs in advancing equitable access to safe, comprehensive health services in urban and rural areas and the reduction of inequities, including with respect to residence, gender, education and socioeconomic position, as well as their role in gaining the trust and engagement of the communities served” [17]. The Assembly also encouraged improved education, retention, management, and remuneration of CHWs, to improve access to health services [17].

In Cameroon, as in most low- and middle-income countries, CHWs are important for the health system [18]. They can fill gaps in the healthcare infrastructure or professional hierarchy and can also enhance health system activities with their cultural and community knowledge [19]. CHWs do not usually have any formal medical education but are effective in delivering preventive or curative health services in diverse contexts [20, 21]. Their usual roles in cervical cancer screening programs are to increase the screening coverage, to educate women about the disease and to manage or assist with the screening and follow up [22].

Retention of CHWs in health programs is, however, a challenge [23, 24]. Ongoing and long-lasting collaboration between CHWs and healthcare professionals is beneficial for both workers and healthcare programs. It ensures continuity between CHWs and the community, maintains their practical and theoretical knowledge and experience, and avoids having to recruit and train new CHWs [24, 25]. It also helps to optimize their performance and implement thriving health programs [26, 27].

The motivation, retention and efficacy of CHWs has been a major concern and subject of research in the last decades. As the success of programs such as the Dschang’s cervical cancer screening programs depends partly on CHWs, the main objective of this study was to identify key factors affecting their motivation. The underlying aim of the study was to facilitate CHWs’ retention by suggesting interventions to address motivational factors.

## Methods

### Study setting

**Fig. 1:**
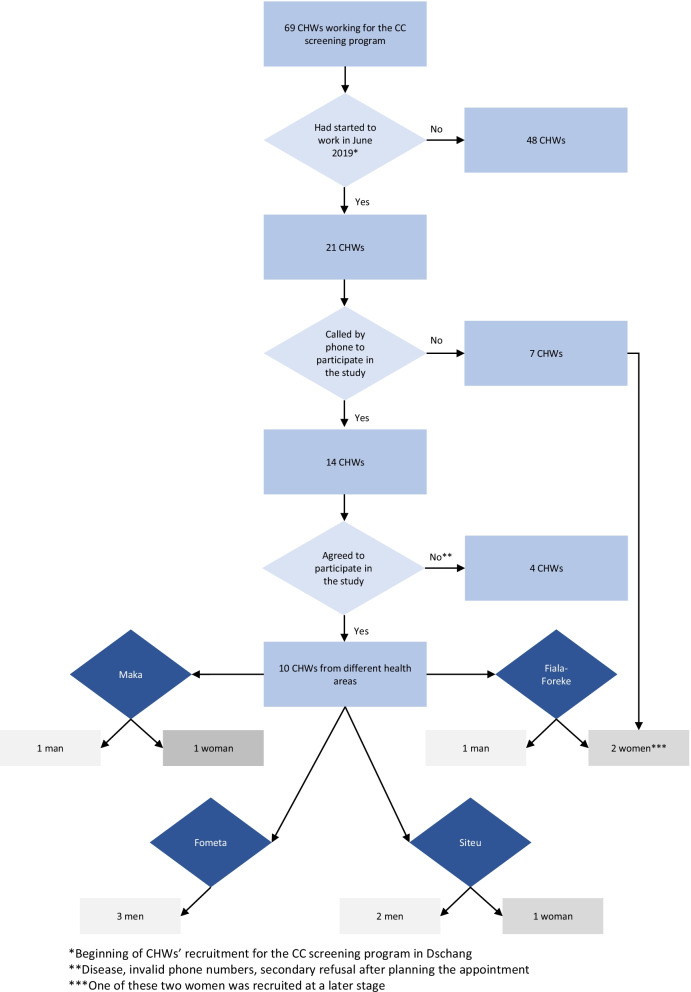
Selection process of the CHWs participating in the study

The cervical cancer screening program in Dschang, Western Cameroon, was initiated in 2015 as part of a collaboration between the Cameroon Ministry of Public Health, the Dschang District Hospital, and the University Hospitals of Geneva [28]. The program targets all women living in the district aged 30–49 years which represents approximately nearly a quarter of population. Since 2018, the 3T strategy has been used, with HPV self-sampling followed by visual assessment for triage of HPV-positive women and treatment by thermal ablation if required. Participants are not charged for these services. CHWs have been included in the program since June 2019, to increase screening uptake. In total, 69 CHWs were recruited on a voluntary basis at the Dschang district level (see Fig. 1) between June 2019 and February 2020. Their main duties are to inform women, their partners, and relatives about cervical cancer and how to prevent it. They also provide information about the cervical cancer screening program and encourage women to attend for screening at the Dschang District Hospital. To understand the effect of CHWs’ information, they distribute tickets with their name to the women and receive for every woman attending the cervical cancer screening program a remuneration.

### Study design

The study used a qualitative design with in-depth face-to-face interviews using a semi-structured interview guide with open-ended questions. The qualitative phenomenological research methodology was chosen as the most appropriate way to gain insights into factors facilitating and impeding motivation among CHWs [29]. The pretested interview guide was developed based on the literature on factors affecting motivation in the target group. It was divided into three categories and CHWs were interviewed about (1) their motivation; (2) the techniques used to recruit women; and (3) any general remarks about the screening program. Every CHW also completed a short questionnaire including socio-demographic questions (such as sex, age and profession).

### Data collection

In total, 11 individual interviews were conducted during July and August 2020. The interviews were held in French and continued until saturation was reached. The interviewer was a female Cameroonian anthropologist with expertise in qualitative research (ADM). Each interview lasted approximately 45 min in a private setting; 10 of the interviews took place in the District Hospital of Dschang and one at the CHW’s home because of sickness. The workers received a snack to thank them for their participation and were compensated for their travel to the hospital if necessary.

### Recruitment of study participants

All CHWs who had been working for the screening program since June 2019 and who had attended a basic theoretical and practical training about screening in October 2019 were eligible to participate. The inclusion criterion of at least 12 months’ experience was based on the hypothesis that work experience prior to the COVID-19 pandemic, which had a considerable effect on the program, would facilitate the evaluation of factors positively or negatively affecting motivation [30].

In total, 21 out of 69 CHWs were eligible to participate. Saturation was expected to be reached after approximately 10 interviews. We therefore initially contacted 14 CHWs by phone and invited them to participate in the study. Four out of these 14 did not participate because of illness (one), invalid phone numbers (two) and secondary refusal (one). More male workers initially accepted, and we therefore invited an additional female worker to ensure a more sex-balanced sample. Selection of participants is summarized in Fig. 1.

### Data analysis

All individual interviews were audio-recorded with the written consent of each participant. Interviews were transcribed verbatim in French and imported to the ATLAS.ti version 8.4.5 software (https://atlasti.com), to allow storing, coding, and management of data. Data were analysed thematically [31] and the study was reported in accordance with the Standards for Reporting Qualitative Research (SRQR) [32].

We used a hybrid approach of deductive (theory-driven) and inductive (data-driven) coding [33]. Two researchers (PH and ADM) independently read the transcripts and coded deductively using the initial codebook of theory-driven codes. New emerging inductive codes suggested by each researcher were discussed and included if both researchers agreed. Once coding was completed, the researchers compared their work to ensure validity. Quotations from the interviews were then translated into English. We developed a conceptual framework based on the literature on motivation of CHWs [34] and public sector healthcare workers [35]. This helped us to organize and illustrate the results and apply social science theories about work motivation to the medical domain.

## Results

### Socio-demographic characteristics

**Fig. 2:**
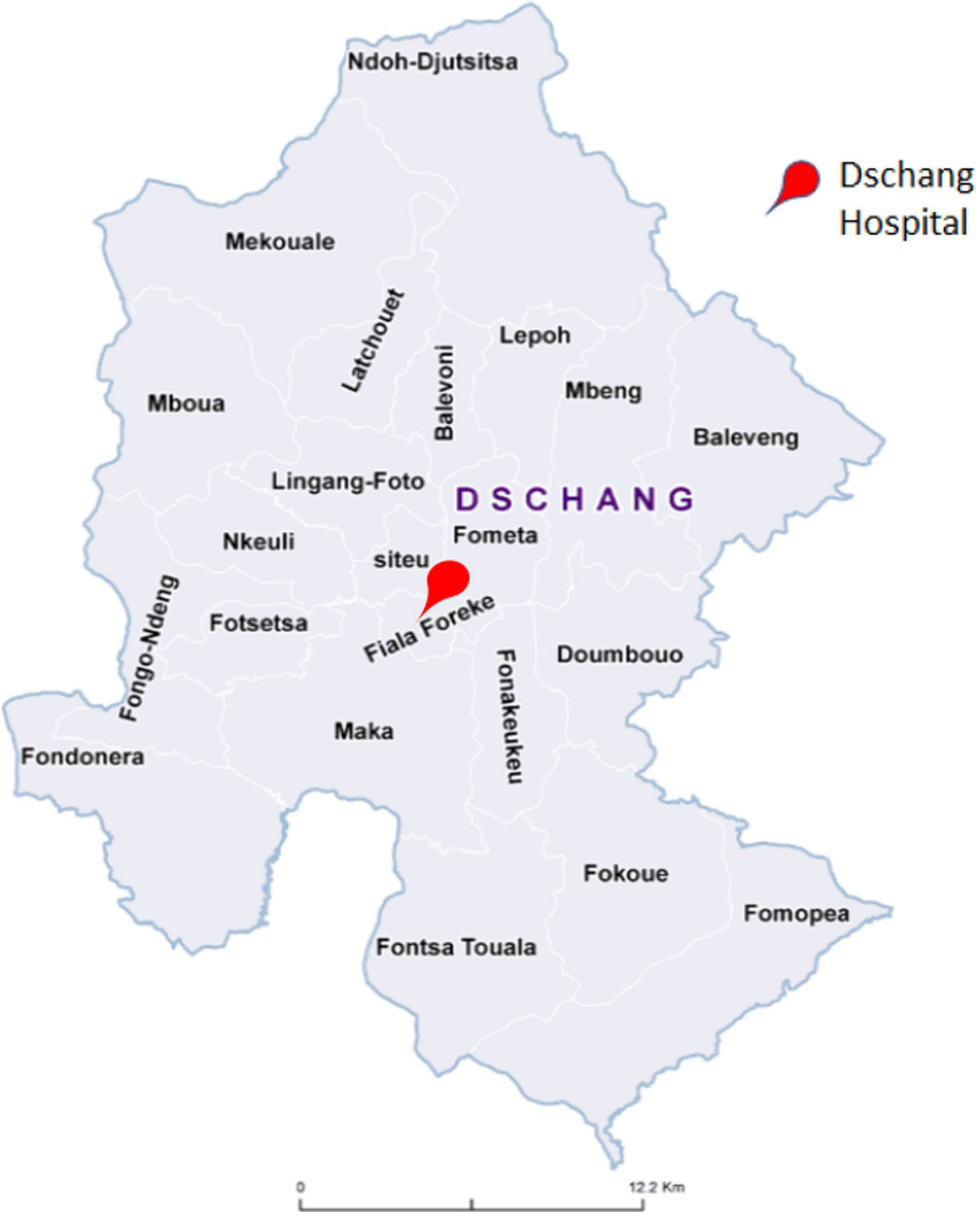
Map of the health areas of the District of Dschang, West Cameroon, modified from Ministère de la Santé Publique du Cameroun (https://dhis-minsante-cm.org/portal/), used from a publication with permission of Datchoua Moukam A.M. [16]

Eleven individual interviews with four women and seven men were carried out. The CHWs were working in four different health areas in the district surrounding the Hospital of Dschang (see Fig. 2). Their median age was 50 years, most were married, and most had attended at least secondary education. Full socio-demographic details are shown in Table 1.

**Table 1:** Sociodemographic characteristics of the CHWs

Variable	Frequency (n = 11)	Percent (%)
Health area		
Fometa	3	27.3
Siteu	3	27.3
Fiala-Foreke	3	27.3
Maka	2	18.1
Sex		
Male	7	63.6
Female	4	36.4
Age (years) Median = 50 Range 21–77		
20–30	1	9.2
30–50	5	45.4
50–70	5	45.4
Marital status		
Married	7	63.6
Single	3	27.3
Widowed	1	9.1
Education		
Primary	2	18.2
Secondary	7	63.6
Tertiary	2	18.2

### Factors affecting motivation of CHWs

**Fig. 3:**
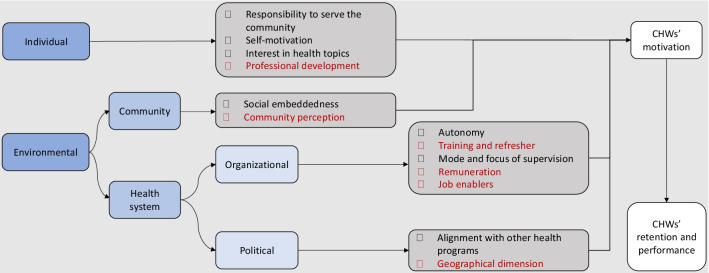
Summary of factors affecting the motivation of CHWs, shown within a conceptual framework. The red text shows the factors affecting motivation that may be altered by key interventions

Factors positively or negatively affecting motivation among CHWs were classified into categories following a conceptual framework adapted from Gopalan et al. [34] and Franco et al. [35] (see Fig. 3). The first category is related to individual-level factors and the second describes environmental factors. Individual factors included all factors affecting individuals, or their beliefs, feelings and aspirations. The environmental factors are external but influence individual behaviour. They include the community and the healthcare system.

### Individual-level factors

The most important factors at individual level were a feeling of responsibility for the community, intrinsic motivation, and interest in health, combined with a desire for professional development.

The *feeling of responsibility* to serve the community and improve the health of community members was perceived unanimously as one of the main facilitators of motivation. Spreading information about cervical cancer screening was seen as both a duty and a normal act of altruism. However, it was also something in which workers took pride. A worker from Fometa explained:

“That's my main motivation, to help my people. My aim was to eradicate this cancer in my community. If I’m a nurse, if I’m a community health worker, and after five years there are still people suffering from cancer, I’d be ashamed. My motivation was to save.” (Fometa01).

*Intrinsic motivation* or *self-motivation* also played a major role. CHWs often also had full-time jobs, and their work for the screening program was an additional voluntary task. However, several mentioned that self-efficacy was an important facilitator for their intrinsic motivation, which was supported by the trust received by the community. A worker from Fiala-Foreke said:

“You accepted of your own free will. When it’s will, nothing can stop you. It’s said: ‘with a valiant heart nothing is impossible’. It’s personal will.” (Fiala-Foreke05)

Several workers noted an important difference between the number of distributed tickets and the actual number of women attending the screening program. This had a negative effect on their intrinsic motivation. A worker from Siteu commented:

“What we’re told is that sometimes they don't come to the hospital. It's very complicated. If you give [the ticket] and they throw it away, how is anyone going to know?” (Siteu09)

The third important factor positively associated with motivation was *interest in topics around health* combined with possible future job opportunities or networks*.* Some of the younger CHWs hoped that their voluntary work would help them to *progress in their career* and become formal health professionals such as qualified nurses. A young worker from Fiala-Foreke explained:

“It has always been my dream to work for the campaign. I hope that in the future I will have the opportunity to maybe find a job in healthcare.” (Fiala-Foreke07)

Workers also saw the international collaboration with the University Hospitals of Geneva as an opportunity to build a *professional network* inside and outside the community. A worker from Fometa reported:

“It’s the staff that’s important. It's having a lot of people, the relationship is more important to me than money. Because I've already had to talk to white women, after three months they leave, there's already my name in Geneva, you came, you called me, that means that it stays in your memory.” (Fometa02).

### Environmental factors

The environmental side can be divided into the community side and the healthcare side.

### Community factors

The two main positive motivators related to the community were social embeddedness and higher social status inside the community. Workers reported that their *social embeddedness* facilitated their interactions inside their community because they know the cultural codes, values, and traditions. Most workers confirmed that recruitment was easier among women from their own community. A male worker from Maka explained:

“For me it's ok because in my health area everyone knows me, they know that I'm their health worker. … And as they already know me, contact is easy.” (Maka03)

Knowing the community also helped workers to engage with people who could gain women’s trust and facilitate the recruitment process. Several workers mentioned involving husbands, the Chief of the village, priests or women who had already undergone screening to help them with recruitment. CHWs’ individual characteristics were also important in the recruitment process. They emphasized that their age or sex influenced their interactions during recruitment. For example, female workers felt more comfortable than their male colleagues in talking about women’s sexuality. However, older workers seemed to carry more weight than their younger peers. Both male and younger workers felt that their status as CHWs helped them to counteract a possible disadvantage.

One of the most important factors affecting motivation was *acknowledgement by the community* and perception of *higher social status*. Workers highlighted that their position was seen as a privilege because they were designated by the so-called “Chief of the health area”^1^. A worker from Fiala-Foreke stated:

“Not everyone can be a health worker. It gives you a bit of respect, you play a role in society.” (Fiala-Foreke07)

CHWs also commented that they valued being exempt from certain common tasks such as cleaning the public areas. They also found that they could ask for services more easily (e.g., help for funerals). However, some noted that their relationships with community members could also be negatively affected by their work. They found that if they insisted that screening was important, women who did not want to attend often perceived them in a more negative way. A worker from Siteu declared:

“It’s a disgrace because when someone doesn't want to, you are like poison to them, an enemy.” (Siteu10)

### Health system factors

Health system factors may be micro or macro level. The micro level is directly linked to the organization of the screening program (the organizational side), and can be often improved directly by interventions inside the program. Macro-level factors include political structures, which are more difficult for health programs to address directly.

### Political factors

The two main political factors mentioned by CHWs were a lack of alignment with other healthcare programs and geographical issues. On *alignment with other existing health programs*, a worker from Fometa explained:

“[I miss out sometimes] because I campaign for this program. Lately there has been the distribution of Mectizan^2^ and some things that I didn't see because I was working on this program.” (Fometa02)

Some workers suggested that local health programs should be coordinated. They felt that once they had gained the trust and respect of the population, they could talk easily about different health subjects with members of the community. They also felt that this would be a better use of resources (e.g. training, shared medical material).

A second important demotivator was the *workload* related to physical capacity. CHWs usually had to walk to many places (e.g., women’s meetings in villages, door-to-door home visits, markets, churches), and older workers found this tiring. One of them said:

“With fatigue it's not easy. The young people still have very strong bones and can take responsibility … [but for us] to walk is too much work. Because it's when you walk a lot that you find what you're looking for. If you only walk a little, you won't find it. You have to walk a lot to find it.” (Siteu09).

### Organizational factors

The most important organizational factors that were positively associated with motivation were autonomy, training, and supervision. The remuneration scheme and a shortage of supporting material were perceived as demotivators.

All CHWs appreciated their *autonomy* in the program because they could choose when and for how long they wanted to work. This perceived feeling of confidence was an important contributor to intrinsic motivation, as a worker from Maka affirmed:

“Monday, Tuesday to Friday. It’s individual, I go door-to-door according to my availability.” (Maka03)

All CHWs appreciated the opportunity to attend *training*. Both basic theoretical and practical training were positively perceived and refresher sessions were suggested. A worker from Siteu said:

“Even if it’s only once or twice a year because it helps you to recall. After you have done a training course, as you work, there are times when you forget certain ideas.” (Siteu10)

Close *supervision and support* from the doctors working in the screening program were perceived as an important motivator. Phone calls to discuss CHW’s performance (average number of women recruited per month) were regarded as helpful because they showed the workers their value in the program. These calls also meant that workers could share any questions and increase their knowledge by exchanging information with healthcare professionals. This made them feel valuable partners and elements of the healthcare system. Several workers felt reassured that they could refer to their supervisors if they faced difficulties convincing women and get advice about recruitment techniques. A female worker from Maka commented:

“Because … there are some cases of refusal, but when we come to see him [a doctor in the screening program], he gives us even more ideas to go and convince the person.” (Maka04)

However, one of the most important factors negatively influencing motivation emphasized by all CHWs was the method of *remuneration*. Workers only received money if women they recruited attended the screening program. They therefore perceived that their work trying to inform women about the program was not valued sufficiently. They suggested that they should be paid for the time spent recruiting women because this would also value their work with women who eventually decided not to attend screening. A worker from Fometa affirmed:

“They say ‘do this’ for one week and they pay me for that week and wait to see if I can come back, and they call me for other neighbourhoods.” (Fometa06)

Financial remuneration was seen as important, but most of the CHWs stated that they would continue to recruit women from easily accessible villages or among family and friends even if they were not being paid. They also noted that the remuneration was often used to pay for transport to and from remote areas. They knew that a woman living further away from the hospital was less likely to come for screening and that they would consequently gain no money.

Another important barrier was the *lack of material* such as umbrellas or boots to help them reach women during the rainy season. They also suggested that it would be helpful to have phone cards to call women before travelling. Some suggested that uniform T-shirts or badges would be a helpful way of increasing their recognition as part of the screening program. A worker from Fiala-Foreke explained:

“Visibility on the field is also needed. That’s to say either shirts or badges [to identify us as part of the program].” (Fiala-Foreke11)

Workers also suggested that posters and medical kits would facilitate their work and that megaphones would support recruitment in public areas.

## Discussion

The objectives of this study were to evaluate factors affecting the motivation of CHWs, and to suggest interventions to help retain their involvement in Dschang’s cervical cancer screening program. Importantly, those factors were mainly environmental (see Fig. 3) and can therefore be directly targeted by healthcare professionals and public health leaders. This section reviews the main findings in the context of the current international literature, particularly on interventions used to improve programs in similar settings.

### Training and supervision

All CHWs perceived training as an important source of encouragement. A qualitative study by Geldsetzer et al. [36] among 54 CHWs in Swaziland showed that training was seen as an opportunity to improve their work quality and performance. This observation was confirmed by our study, where all participants asked for refresher training to keep their knowledge up to date. Previous studies [21, 37, 38] have also demonstrated that training increases CHWs’ self-confidence and self-efficacy.

Especially younger CHWs mentioned aspirations for career development as an important motivator. Previous studies [24, 37, 39] have found that CHWs are motivated when they feel that they are making progress and have stressed the importance of personal achievement. Another closely related issue is workers’ appreciation of supervision and support from healthcare professionals. Exchanging information and discussing difficulties encountered while recruiting women for screening were perceived as important factors to improve self-assurance and performance among CHWs [40].

Health programs should therefore evaluate how training and supervision can support CHWs to improve performance. They should also consider some form of professional development, such as giving workers more responsibility (e.g., being responsible for a group of CHWs or assisting with screening and follow up). They may even offer opportunities to attend formal health professional training programs.

### Community perception

Previous studies have highlighted that working as a CHW may increase social status [20, 37, 41]. A qualitative study by Greenspan et al. [42] in Tanzania showed that this was mainly expressed by increased respect and recognition, and provision of small services by the community. This observation was confirmed in our study. However, some workers feared that there might be a negative impact on their status in the community when they had difficulties convincing women to attend screening. An Indian qualitative study [20] found that training can help CHWs to better cope with this negative experience or the disappointment of refusal and encourage peer support. Equivalent interventions have also been proposed in other studies [39, 43, 44].

Training and supervision can improve performance and work quality. Our results suggest that it may be helpful to provide training to strengthen CHWs’ communication skills. A recent qualitative study [16] at the Dschang District Hospital explored women’s barriers to using the cervical cancer screening program. It found that some women perceived healthcare providers as disrespectful because of the way that they communicated. Workers in our qualitative study reported negative experiences when trying to convince women who refused to attend screening. It is therefore important to understand their communication techniques following refusals. A recent cross-sectional study [45] in seven African countries reported a positive correlation between patient experience and healthcare providers’ level of education. CHWs often have lower levels of education, and communication skills and techniques should therefore be included in their training and supervision.

### Remuneration and supporting material

Previous studies [20, 24, 46] have highlighted the importance of financial motivation and low amounts of payment are often described as a hindrance to motivation [37, 39]. However, the CHWs in Dschang mainly perceived the current payment scheme as not appropriate. They would prefer to be paid for the hours they spent recruiting women (rather than the number of women attending screening). However, they reported a wide range of working hours per week, which did not always correlate with the number of women recruited. This is consistent with a previous study [47] that made a comparative analysis of qualitative studies in Bangladesh, Ethiopia, Kenya, Indonesia, Malawi and Mozambique. That study found that if the monetary rewards received by CHWs did not match their efforts, their motivation was negatively affected.

We recommend that CHWs in the screening program should understand the remuneration system before being engaged. It should also be clear that their work should be considered as a side-job, so that they can adapt their expectations. On the other side, changes in the remuneration mode and amount should also be considered and evaluated within the program structure.

CHWs mentioned several factors that could facilitate their work recruiting women for screening. These included phone cards to reach women, or umbrellas and boots for the rainy season. Other factors such as wearing uniform shirts or badges would increase the feeling of belonging to the program and anchor their position within the community. This is consistent with previous studies that confirmed the association between these factors and trust in CHWs’ ability to do the job [19, 21, 24]. We suggest that the screening program should evaluate the potential to provide material to reinforce CHWs’ credibility within the community and improve their performance.

CHWs also need support with transportation. Transport to remote areas was a major obstacle, especially for older workers, or those with limited physical capacity. A mixed method study from Tripathy in India [37] showed that older CHWs were less motivated than their younger colleagues, partly because of the heavy workload. However, it is important to retain older CHWs because they are very respected in the community. Possible solutions are to compensate for transport depending on the distance to the District Hospital of Dschang. Other factors such as phone credit could help workers to avoid unnecessary journeys.

### Other existing health programs

The CHWs suggested coordinating different local health programs. This is consistent with a study by De Neve et al. [48] in four Southern African countries. The study found that it was helpful to align healthcare programs in the same region that were delivering HIV services and employing CHWs. Harmonization can help to avoid duplication of services and confusion in responsibilities, save resources and strengthen the role of CHWs. This is potentially applicable in our setting. However, alignment of healthcare programs requires involvement of political leaders, and therefore cannot be addressed directly by the screening program alone.

### Study limitations and strengths

To our knowledge, this study is one of the first in Cameroon to examine factors influencing CHWs’ motivation from a qualitative perspective. However, it had several limitations. First, interviews were led by a local anthropologist in a private setting, but interviewer bias cannot be excluded. It is also possible that interviewees answered questions to satisfy the interviewer, rather than sharing their own opinions. Second, CHWs have only been involved in the cervical cancer screening program since June 2019, so they have relatively little work experience, and the program has been disrupted by the COVID-19 pandemic. Other motivators and demotivators could emerge in future as the program develop. Lastly, as with other qualitative studies, the aim is not to generalize the findings. In counterbalance, we took several measures to eliminate or reduce bias, including developing the interview guide together, and carrying out independent coding followed by discussion among the two main researchers. Saturation was reached in the 11 interviews and the findings on factors affecting CHWs’ motivation encountered were consistent within the literature. We therefore consider that our findings are likely to be generalizable to other similar settings.

## Conclusion

The results of the study are very important because the inclusion of CHWs in healthcare programs is crucial, especially in low- and middle-income countries. Studies like this increase knowledge how to improve workers’ performance and about work quality inside programs. High motivation is strongly associated with improved retention and performance of CHWs in healthcare programs. It is therefore important to address factors that negatively affect motivation. We recommend several key interventions covering both monetary and non-monetary incentives, including:

(i) increased opportunities to attend training and formal supervision;
(ii) regular evaluation of the mode and amount of remuneration by workload; and
(iii) provision of supporting material to facilitate the role of CHWs in the community and address travel distance.

Further qualitative and quantitative research is needed to develop a better understanding of the required training and supervision components (such as communication skills or supervision groups) and establish how interventions can be linked to the performance of CHWs and screening uptake of women.

## Data Availability

The verbatim transcripts are not publicly available to protect data privacy. The interview guide and summaries of transcripts, including categories and codes, are available on request from the corresponding author.

## Abbreviations

HPV: Human papillomavirus
WHO: World Health Organization
CHWs: Community healthcare workers

## Footnotes

1 The health district is subdivided into health areas. Each health area is made up of a village or set of villages with a total population of 5000 to 10,000 inhabitants, with a well-defined territory, a manager or chief and served by a health centre that is responsible for health activities.

2 Mectizan is the commercial name for ivermectin, an antiparasitic drug.
